# Knowledge, attitudes and practices towards menopause among Congolese middle-aged and postmenopausal women

**DOI:** 10.11604/pamj.2021.38.315.20342

**Published:** 2021-03-30

**Authors:** Sifa Marie Joelle Muchanga, Anyeke Louise Lumumba, Bebele Jean Patrice Kamba, Etongola Papy Mbelambela, Antonio Fredelindo Villanueva, Marlinang Diarta Siburian, Rahma Rashid Tozin

**Affiliations:** 1 Department of Obstetrics and Gynaecology, Medical Faculty, University of Kinshasa, Kinshasa, Democratic Republic of the Congo,; 2 Department of Environmental Medicine, Kochi Medical School, Kochi University, Nankoku, Kochi, Japan,; 3 Department of Family and Tropical Medicine, San Lazaro Hospital, Manila, Philippines,; 4 Department of International Trials, Clinchoice, Tokyo, Japan

**Keywords:** Knowledge, attitudes, perceptions, practices, menopause, Congolese

## Abstract

**Introduction:**

with the increase in life expectancy, women will live longer during their postmenopausal period. To improve their quality of life, they should be aware of what challenges they will be facing. This study aimed to evaluate the knowledge, attitudes and practices of middle-aged women towards menopause.

**Methods:**

in this cross-sectional study, data collected using a multistage clustered random sampling from 54 health centres in the Democratic Republic of Congo were used. Participants filled a questionnaire derived from the menopause rating scale and from local beliefs. The knowledge, attitudes and practices towards menopause were evaluated among pre- and postmenopausal women.

**Results:**

of the 353 women, both pre- and postmenopausal women knew the definition of menopause but for the symptoms, postmenopausal women were more informed than premenopausal. For the attitudes and practices towards menopause, while both had equally positive attitudes, the premenopausal women did not know which practice to adopt.

**Conclusion:**

Congolese women had limited knowledge, positive attitudes and unconventional practices towards menopause. Health-care providers, therefore, need to dispense appropriate advice to middle-aged women before the advent of menopause.

## Introduction

Menopause is a natural process of aging defined as a definitive cessation of ovarian follicles activity and consequently, the end of menstruations [1]. This period is critical and complex because the woman is subject to face many challenges due to changes in her physical and emotional life. The way she reacts to and manages the biological, psychological, cultural, and economic changes influences the rest of her life and her attitude in front of menopausal events such as the transition into menopause, midlife, and aging [2]. The attitudes and practices towards menopause have been influenced by a range of beliefs related to sociocultural differences and to the background of the women which may radically affect the menopausal experience and reporting of symptoms [2-5]. While natural menopause is a physiologic process of a woman´s lifecycle, the quality of life during the period from menopausal transition to menopause may vary among countries, within the same country among cultural groups, and even between individual women. They may experience numerous symptoms including somato-vegetative, urogenital and psychological symptoms [6]. Since the total number of postmenopausal women in the world is expected to reach 1.1 billion by the year 2025 [7], and menopause is experienced in a variety of ways by different women, appropriate care and support are needed in order to improve the quality of life of this vulnerable population group. The quality of life in menopausal period is multifactorial and results on the combination of psychosocial, cultural, clinical and environmental factors [2]. It has been demonstrated that knowing the symptoms of perimenopausal transition before their experience can positively influence women´s attitudes during this period [8]. In low and middle-income countries, which is the case for most of sub-Saharan Africa, little attention has been given to women´s knowledge, attitudes and practices towards menopause and related factors [9,10]. The majority of studies have been focusing on the experience and reported symptoms during menopausal transition and menopause [11-15]. Studying menopause and menopause-associated disorders such as osteoporosis, metabolic syndrome, and prehypertension have been the subject of interest for researchers in the Democratic Republic of the Congo (DRC) [16-19]. However, none of them have considered assessing the awareness of the definition, the symptoms, the attitudes, and the practices towards menopause among middle-aged and/or postmenopausal Congolese women. In order to develop an integrated program to fully support and improve the quality of life of these middle-aged women, health care providers need to be aware of how much the women perceive this period of life. As the first step for the development of evidence for policy maker to integrate middle-aged women care, this study aimed to evaluate the knowledge, attitudes and practices towards menopause among a group of Congolese middle-aged women living in Kinshasa.

## Methods

We conducted a cross-sectional exploratory study from February 25 to April 25, 2017 in the city of Kinshasa, the capital of the DRC.

**Sampling procedure and data collection:** we performed a multistage, clustered random sampling in three steps. From the six health districts of Kinshasa, we randomly selected three health zones per health district and then per health zone, we randomly selected three reference health centres. In total, 54 health centres were selected for the study. We administered training to two surveyors per health centre. All women who visited the selected health centres for various reasons during the study period received the explanation about the content of the study from their regular physicians. Then, those aged 40 to 65 years old who agreed to participate in the study were interviewed by trained surveyors. Women less than 40 years and those over 65 years old were excluded from the analysis. The study was approved by the School of Public Health ethical committee of the University of Kinshasa, the Provincial Division of Health, and the Executive board of each health centre in Kinshasa. Data were collected anonymously and all participants provided their written informed consent in accordance with the ethical standards set forth in the Helsinki declaration.

**Survey questionnaire:** we divided the questionnaire into three parts: socio-demographic information, general knowledge on menopause, and attitudes and practices towards menopause. Socio-demographic information comprised of age, parity, profession, ethnicity, education, marital status, and menopausal status. Considering the menopausal status, we defined women as postmenopausal if they had no history of hysterectomy and had cessation of their menstruations for more than one year [20,21]. Women who were still experiencing their menstruations as of the study period were classified as premenopausal. Concerning the general knowledge on menopause, attitudes and practices towards menopause, women were asked to choose “I agree” or “I do not agree” to some questions derived from the literature [4,5,9,22] and from local beliefs. We assessed the knowledge about the definition of menopause by asking each participant if she agreed to the fact that menopause is a definitive cessation of menstruations, a loss of ability to reproduce naturally, and a natural process. Knowledge on menopausal symptoms was evaluated using items included in the Menopause Rating Scale [6]. Second, we assessed the positive attitude towards menopause by asking the following questions: during menopause, is menopause a normal aging process? Are sexual activities possible during menopause? Does menopause add freedom from menstrual bleeding? Does the woman have more value in the society has a mature person? For the negative attitude, does the woman become different? Should the woman change her partner? does the woman lose her womanhood and value in the society (meaning the woman has reached a bad stage of her life)? Is menopause a threatening event? To each question, the participant replied by either “yes”, “no” or “I do not know”. Third, to evaluate the knowledge about which practice to adopt during the postmenopausal period, we asked women to reply to the following questions: should a woman visit the physician when she has her menopause? If she has to choose a treatment which one do you think she may take? This was an open question and participants provided either by comment or one of the following answers: hormono therapy, traditional plants, calcium supplementation, analgesic, the use of saliva for vaginal lubrification.

**Figure 1:**
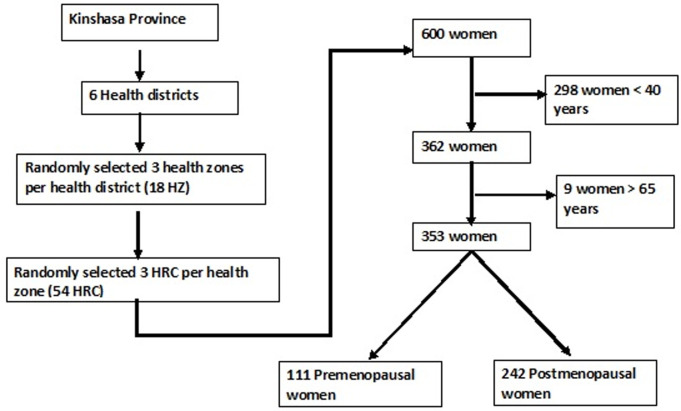
study sampling and flow chart

**Sample size:** during the study period, out of the 660 women who were consecutively recruited from the selected health centres, 298 where aged less than 40 years and 9 were more than 65 years old. After their exclusion, 353 middle-aged women were interviewed. Of the remaining women, 111 (31.4%) were premenopausal and 242 (68.6%) were postmenopausal (Figure 1).

**Statistical analyses:** we summarized quantitative data as mean and standard deviation and qualitative data by proportion. To test the intergroup differences, we performed the chi square test and the unpaired student's t-test respectively for qualitative and quantitative variables. The significance level was set at 0.05. We analysed data using the Stata (STATACORP, USA) Software version 15.0 for Windows.

## Results

**General characteristics:** as reported in Table 1, the average age of the study population was 50 years, postmenopausal being older than premenopausal women (p<0.001). They also had lower education level, had more widows in their group and were unemployed as compared to the premenopausal women (p<0.001).

**Table 1:** general characteristics of the study population

Characteristics	Total population (n=353)	Premenopausal (n=111)	Postmenopausal (n=242)
**Age, y; Mean (SD)**	50.9 (7.4)	43.7(3.2)	54.3(6.4) ***
**Marital status**			
Single	11(3.1)	8(7.2) ***	3(1.2)
Married	253(71.7)	97(87.4) ***	156(64.5)
Divorced	30(8.5)	3(2.7) ***	27(11.2)
Widow	47(13.3)	1(0.9)	46(19.0) ***
Others	12(3.4)	2(1.8)	10(4.1) ***
Parity	5.4(2.5)	4.3(2.2)	5.9(2.5)
**Ethnicity**			
Swahili	60 (17)	16(14.4)	44(18.2)
Lingala	30(8.5)	11(9.9)	19(7.9)
Luba	70(19.8)	26(23.4)	44(18.2)
Kongo	147(41.6)	43(38.7)	104(43.0)
Missing	46(13.0)	15(13.5)	31(12.8)
**Education**			
No education	16(4.5)	4(3.6)	12(5.0) ***
Primary	87(24.7)	19(17.1)	68(28.1) ***
Secondary	197(55.8)	56(50.4)	141(58.3) ***
Tertiary	53(15.0)	32(28.8) ***	21(8.7)
**Profession**			
Unemployed	282(79.9)	81(73.0)	201(83.1) *
Student	70(19.8)	29(26.1) *	41(17.0)
Employed	1(0.3)	1(0.9)	0

**Knowledge of menopause and related symptoms:** in general, both premenopausal and postmenopausal women knew at least one component of the menopause definition except for the cessation of menstruations for which there was a difference in favour of postmenopausal women (p<0.01). With regard to the menopause-related symptoms, the most reported symptoms by the two groups were hot flushes and night sweats but upon further comparison, premenopausal women had a general lack of knowledge on almost all symptoms except for dryness of vagina. For other symptoms, they were unaware of their existence, including sleeping problems, depressive mood, anxiety, physical and mental exhaustion, sexual problems, joint and muscular discomfort, and weight gain. Both pre- and post-menopausal women did not have knowledge about bladder problems and anxiety. The results are represented in Table 2.

**Table 2:** knowledge on menopause and related symptoms

Characteristics	Total population (n=353)	Premenopausal (n=111)	Postmenopausal (n=242)
**Definition**			
Cessation of menstruations	349(98.9)	107(96.4)	242(100) **
Definitive process	215(60.91)	60(54.1)	155(64.1)
Natural process	327(92.6)	101(91.0)	226(93.39)
Cessation of fertility	336(95.18)	106(95.1)	230(95.0)
Hot flushes, night sweats	130(36.8)	3(2.7)	127(52.5) ***
Heart discomfort	6(1.7)	0	6(2.48)
Sleep problems	13(3.7)	0	13(5.4) *
Depressive mood	61(17.3)	1(0.9)	60(24.8) ***
Irritability	10(2.8)	0	10(4.1) *
Anxiety	0	0	0
Physical and mental exhaustion	27(7.7)	0	27(11.2) ***
Sexual problems	19(5.4)	0	19(7.9) **
Bladder problems	0	0	0
Dryness of vagina	19(5.4)	1(0.9)	18(7.4)
Joint and muscular discomfort	63(17.9)	0	63(26.0) ***
Weight gain	32(9.1)	0	32(13.2) ***

* p<0.05; **p<0.01; ***p<0.001

**Attitudes towards menopause:** concerning the attitude towards menopause, as reported in Table 3, both premenopausal and postmenopausal women presented equal views whether positive or negative. Fifty-four percent of the study population did not know that menopause is a normal ageing process. In addition, 26% of them reported that women lose their womanhood during menopause and 18% said that menopause is a threatening event.

**Table 3:** attitude towards menopause

Characteristics	Total population (n=353)	Premenopausal (n=111)	Postmenopausal (n=242)
**Positive**			
Menopause is a normal ageing process			
Yes	111(31.4)	29(26.1)	82(33.9)
No	51(14.5)	18(16.2)	33(13.6)
I do not know	191(54.1)	64(57.7)	127(52.5)
**Sexual activities are possible**			
Yes	292(82.72)	96(86.5)	196(81.0)
No	46(13.0)	10(9.0)	36(14.9)
I do not know	15(4.3)	5(4.5)	10(4.1)
**Menopause add the woman's freedom**			
Yes	179(50.7)	54(48.7)	125(51.7)
No	84(23.8)	26(23.4)	58(24.0)
I do not know	90(24.4)	31(27.9)	59(24.4)
**Woman has more value in the society**			
Yes	167(47.3)	58(52.3)	109(45.0)
No	81(23.0)	27(24.3)	54(22.3)
I do not know	105(29.8)	26(23.4)	79(32.6)
**Negative**			
**The woman becomes different**			
Yes	90(25.5)	33(29.7)	57(23.6)
No	103(29.2)	29(26.1)	74(30.6)
I do not know	160(45.3)	49(44.1)	111(45.9)
**The woman should change the partner**			
Yes	50(14.2)	18(16.2)	32(13.2)
No	186(52.7)	66(59.5)	120(49.6)
I do not know	117(33.1)	27(24.3)	90(37.2)
**The woman loses her womanhood**			
Yes	92(26.1)	28(25.2)	64(26.5)
No	53(15.0)	11(9.9)	42(17.4)
I do not know	208(58.9)	72(64.9)	136(56.2)
**Menopause is a threatening event**			
Yes	65(18.4)	17(15.3)	48(19.8)
No	29(8.2)	7(6.3)	22(9.1)
I do not know	259(73.4)	87(78.4)	172(71.1)

**Knowledge about the practices towards menopause and its treatment:** as presented in Table 2, none of the premenopausal women knew about which practices to adopt towards menopause and its related symptoms. For postmenopausal women, 19% of them reported that the physician should be consulted. Regarding the treatment, in general, their knowledge about available treatment options during menopause varies from 0.8% to 4.6%; two of the postmenopausal women (0.8%) reported that saliva can be used for vaginal lubrication (Table 4).

**Table 4:** practice towards menopause and menopausal treatment

Characteristics	Total population (n=353)	Premenopausal (n=111)	Postmenopausal (n=242)
The woman should visit the physician	47(13.3)	0	47(19.42)
**Which treatment to take?**			
Hormonotherapy	11(3.1)	0	11(4.6)
Traditional plants	2(0.6)	0	2(0.8)
Calcium supplementation	2(0.6)	0	2(0.8)
Analgesic	9(2.6)	0	9(3.7)
Use of saliva for vaginal lubrication	2(0.6)	0	2(0.8)

## Discussion

To enhance the overall quality of life of women during transition to menopause and during post-menopausal period, it is important for the woman to know necessary information on what menopause is and how to deal with its related problems. Clinicians are recommended to interact with middle-aged women in order to give them advice on how to manage this crucial period of their lives [23]. This study aimed to evaluate the knowledge, attitudes and practices towards menopause among middle-aged Congolese women. An overwhelming 99% of participating women knew about the definition of menopause as a cessation of menstruations, but only 61% of them reported that menopause is a definitive process. As for menopause-related symptoms, except for “hot flashes and sweating” which was reported at 53%, only less than half of postmenopausal women were aware of other symptoms. None of them knew about the existence of anxiety and bladder problems. Very few premenopausal women reported having knowledge on symptoms except for hot flushes and sweating, depressive mood, and dryness of vagina. Our results are similar to those reported in Ecuador by Leon *et al*. in which 60.2% of participants correctly defined menopause and less than 50% considered having enough information regarding menopause [4]. In Taiwan, Pan *et al*. also reported that only 53% of participants could give the correct definition of menopause [24]. Vasomotor symptoms dominated by hot flushes and sweating followed by joint discomfort are the most commonly reported symptoms of menopause in the literature [12]. However, the pathophysiology of hot flushes is not clearly established. Nevertheless, the ovarian follicular failure accompanied with the oestrogen deprivation state observed during the menopausal transition may be the main cause of vasomotor symptoms and joint pain [13]. The decline in oestrogen leads to an up regulation of serotonin receptors involved in thermoregulation and to the increase in norepinephrine levels [25]. Regarding bone metabolism dysfunction, the decrease of oestrogen and the subsequent increase of follicle-stimulating hormone (FSH) production stimulate bone resorption and consequent bone loss [26].

In general, all participants had a positive attitude towards menopause but only one third described menopause as a normal ageing process while more than half of the population did not know. This situation could reflect the low literacy rate of women in the DRC which is 66.5% for those aged 15 years and older [27]. In addition, 26% of the study population reported that women lose their womanhood during menopause and 18% said that menopause is a threatening event. Overall, the positive perception of menopause found in this study has been previously reported in different settings and populations [4,12,22,28-30]. When we compared the two groups, both premenopausal and postmenopausal women had the similar attitudes towards menopause, whether positive or negative. However, none of the premenopausal women knew which practices are recommended after menopause. The same trend was observed for the large majority of postmenopausal women. Moreover, some postmenopausal women reported unconventional practices such as using saliva for vaginal lubrication. Since the menopause-related symptoms are experienced by all menopausal women regardless of their origin or their economic status, Obermeyer reported in a review that the association between menopausal status and the perception of menopause is not highly significant [3]. Still, women´s personal and environmental influences weigh heavily on their attitudes towards menopause [2].

The lack of knowledge on practices to adopt during the postmenopausal period highlights again the low literacy rate of Congolese women [27]. However, this cannot explain the unconventional practices reported by some women. The environment where traditional beliefs and oral tradition are popular could be one of the vehicles for such attitudes. To the best of our knowledge, this study is the first to evaluate the knowledge about menopause, attitudes and practices towards menopause in both pre-and postmenopausal Congolese women. This study highlights the depth of misinformation on menopause and related events in this sample of middle-aged women. Our study had some limitations that should be addressed. For instance, we did not collect data on the source of information, making it difficult to determine from which source the wrong information was acquired.

Alongside the cross-sectional study design, the random multistage sampling procedure used for data collection makes the results representative of the population of Kinshasa. Longitudinal studies on menopause-related symptoms, treatment, and risk factors concerning negative attitudes and/or unconventional practices should be conducted in order to have a clear understanding on which aspect targeted action can make a difference. Our results can be valuable for clinicians and health providers to understand that the specific population of middle-aged women needs more attention to improve their quality of life during postmenopausal period. Future postmenopausal women need to be prepared in advance for the management of menopause- associated physical and socio-emotional symptoms.

## Conclusion

Congolese women, in general, had limited knowledge of menopause, attitudes and practices to adopt during postmenopausal period. The attitude towards menopause was positive regardless of the menopausal status. As the source of information was not determined and some women reported unconventional practices, the implementation by policy makers of a comprehensive global and strategic educational program targeting middle-aged women possibly via mass media and community leaders such as in churches might be one of the strategies to fill this gap. This study intends to serve as an evidence for planning the improvement of health services targeting middle-aged women in sub-Saharan countries.

### What is known about this topic

- Postmenopausal women are aware of symptoms of menopause;
- Attitudes and practices towards menopause related-events vary by culture and by population;
- Little is known about the premenopausal middle-aged women reaction towards menopause.

### What this study adds

- Both postmenopausal and premenopausal women are aware about menopause-related events but their knowledge is limited and not fully accurate;
- Sometimes their attitudes towards menopause are inappropriate.
